# A Sustainable Substitute for Ivory: the Jarina Seed from the Amazon

**DOI:** 10.1038/srep14387

**Published:** 2015-09-24

**Authors:** Yinghao Chu, Marc A. Meyers A, Bin Wang, Wen Yang, Jae-Young Jung, Carlos F. M. Coimbra

**Affiliations:** 1 Department of Mechanical and Aerospace Engineering, Jacobs School of Engineering, University of California, San Diego, La Jolla, CA 92093, USA

## Abstract

The dried endosperm of the seed of *Phytelephas sp* is widely used for artisanal work in the Amazon region due to its favorable mechanical properties and pleasant appearance that resemble elephant ivory. While the seeds have enjoyed popularity and limited use by selected industries (e.g., military uniform buttons and piano keys) and handicraft applications, little is known about the mechanical properties and structure of this sustainable material. This work is the first to characterize the dried Jarina endosperm and to investigate its functionality as a viable substitute for elephant ivory. Structural analysis of typical seeds reveals the prevalence of tubules that align in rings and radiate from the (usually hollow) core of the seed. This seed, in the absence of a reinforcement structure or mineral phase, possesses mechanical properties slightly inferior to elephant ivory and selected plastics, while retaining the visual appeal of a naturally occurring material. A synthetic structure inspired on the seed is created and suggestions for further development are discussed.

Ivory has been considered as a valuable bio-tissue since prehistoric times. However, excessive trade of elephant ivory has contributed to the decline of elephants, particularly from the three subspecies of African elephants, which are known for their massive tusks. Because African elephants are critical elements of the ecosystem in many African habitats, the depletion of elephant populations is of serious concern. The population of Africa’s forest elephants has shrunk by 60% in the past decade, and there are recent signs of increasing stress on several elephant communities. Most of this stress can be directly linked to poaching for the illegal but highly profitable trade of elephant ivory^1^. Artificial substances like celluloid and other plastics have been used as ivory replacement, but they lack the appeal of naturally occurring fibers and polymers, and their widespread use brings concerns related to the slow degradation rates of plastics in the biosphere. Vegetable ivory (species in the genus *Phytelephas*, also named “Jarina” in the Brazilian Amazon region) resembles closely the appearance of elephant ivory (Fig. 1a), while offering many desirable physical characteristics like biodegradability, conformability, carvability, and other outstanding mechanical properties. The endosperm is a milky liquid, which is usually sun-dried until it turns into a very hard material. Once a popular material in the clothing industry (according to Acosta-Solís, 1948^2^, a large share of the buttons of the U.S. military uniforms were made from it during World War II), the cultivation and trade of Jarina palm seeds have endured periods of complete abandonment in the past few decades. However, in recent years, environmental concerns regarding the biodegradability of plastic commodities have motivated the re-discovery of the Jarina seeds^3^.

The majority of scientific studies relate to the biological taxonomy and chemistry of the Jarina seed. Some have discussed its chemical components, mainly focusing on the hydrolysis and extraction of the mannose^3^,^4^,^5^. The main components of the dried cores are hemicelluloses^3^. When it is hydrolyzed, the major component is mannose, which corresponds to about 86 percent of its weight. Other components include glucose, galactose, arabinose, xylose, and rhamanose^5^.

However, no previous attempt has been carried out to quantitatively study the mechanical properties of the dried Jarina endosperm, which is essential to the full understanding of this material and the promotion of its cultivation and applications. Mechanical failure of the Jarina seed is further studied using a statistical Weibull analysis; its anisotropic behavior is modeled with a stress concentration model. The K-R (fracture toughness as a function of crack extension) curve is obtained to analyze its fracture toughness. These structural and mechanical properties are compared, for the first time, with those of elephant ivory and plastics to inform on its potential as elephant ivory substitute.

We observed a central lacuna located inside each Jarina seed (in Fig. 1a). The density of vegetable ivory is 1.2 ± 0.2 g/cm^3^ (in Table 1), which is lower than that of natural ivory, hippo tooth, antler bone, and celluloid^6^.

X-ray tomography (Fig. 1b–d) shows that the vegetable ivory is porous. There are cylindrical primary tubules from which secondary tubules radiate. The primary tubules show varying diameters (25–50 μm), and are arranged in arrays forming concentric rings, in which the longitudinal axes of these primary tubules are perpendicular to the ring radius (Fig. 1b,c); the ring thickness corresponds to the tubule length, ~100 μm). The primary tubules are distributed in hexagonal arrangement in the transverse section (shown in Fig. 1f), indicating that the structure of vegetable ivory is similar to a lotus-type porous material.

We represented the Jarina nut in a 3-D spherical coordinate system (shown in Fig. 1e). This radial distribution of tubules might result in anisotropic mechanical behavior. We defined a transverse plane (TP) and a radial plane (RP) based on the orientations of the internal tubules: TP is perpendicular to the longitudinal axis of the tubules, and RP is parallel to it. Optical microscopic images for both planes are shown in the Fig. 1f,g; the hexagonal distribution of primary tubules is readily seen.

Under UHR SEM (×40,000), the structures, (e.g. mineral phase or reinforcement fiber) characteristic of ivory at different length scales (ranging from nanometers to micrometers), are absent. The Jarina seed is dried from its emulsion state under the sunlight for periods that can extend to a few weeks. Considering that hemicellulose is amorphous, we suggest that the seed is a single-phase material, and any anisotropic behavior is solely due to the orientation of tubules.

Results of tensile tests are provided in Table 1. The strain to failure is 0.03 ± 0.01. The average Young’s modulus is 1.5 ± 0.5 GPa, consistent with a large variation characteristic of biological materials^7^. Mechanical properties of elephant ivory and celluloid are provided in Table 1 for comparison. Vegetable ivory shows a Young’s modulus and a work to fracture that are on the same order of magnitude of elephant ivory. They are also comparable to the plastics like celluloid, which has been first widely used as an ivory replacement.

The Weibull analysis provides a continuous probability distribution that has been successfully employed for statistical analysis of the results for a number of biological materials^7^,^8^,^9^. It assesses the distribution of properties through a function, 
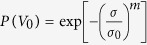
, where *P(V*_*0*_) is the probability of survival at stress *σ*, *σ*_*0*_ is a characteristic stress, and m is the Weibull modulus that measures the variability of strength (lower value means higher variability)^7^,^8^,^10^. Weibull analysis on the tensile testing results shows that m is equal to 2.2, which confirms that the material has a considerable variability in strength, typical for biological materials (metals show m as 10–20, ceramics around 3)^10^. The stress at 50% probability of failure is 26 MPa.

The microhardness data are presented in Table 2 and compared to that of elephant ivory^11^. Microhardness in both directions is about one third of those of elephant ivory. Results of nanoindentation tests are shown in Fig. 2a: 378 MPa and 385 MPa on transverse and radial planes, respectively. The elastic moduli measured from the nanoindentation tests are 7.8 GPa and 8.2 GPa, respectively. The nanohardness is consistently higher than the microhardness because: (1) the tubules at micro-scale can affect the microhardness values, but not the nanohardness since these tests are done in the space between them; (2) nanoindentation excludes the effects of micro-scale cracks, and other structural defects and the values are that for an ideal material. Unlike the microhardness, nanohardness does not show significant anisotropy. This is consistent with the observation that the nanoindentation is sufficiently small to avoid the interference from tubules; thus, the nanoindentation results further confirm the assumption that the vegetable ivory is a single phase material and that the anisotropic behavior is only caused by the distribution of tubules.

Two compressive loading directions were applied: normal to the transverse plane (defined as TP orientation) and the radial plane (defined as RP orientation). The compression curves of the samples exhibit three stages: elastic deformation, plastic deformation, and failure (without densification). Anisotropic behavior is observed: TP orientation shows a higher elastic modulus but lower strain to failure than RP. The strain to failure ranges from 0.2 to 0.35 for TP and from 0.18 to 0.4 for RP. The results of the tests are presented in Table 3.

A stress concentration model is applied to explain the anisotropy. Schematics of compressive loading perpendicular and along to the tubules are shown in Fig. 2b. The lower strength of the RP orientation test results is caused by the higher stress concentration when loading is applied perpendicular to the cylindrical tubules^12^; this stress concentration is lower if loading is aligned with the tubules^13^. Therefore, vegetable ivory is more resistant to stress normal to the TP than normal to the RP. The cracks tend to propagate along the RP rather than the TP. Compressive behavior from both orientations was evaluated through Weibull analysis (Fig. 2c,d). The m for TP (3.56) is significantly higher than m for RP (2.76). The stress at 50% probability of failure for loading perpendicular to the TP is 160 MPa and for loading perpendicular to the RP is 140 MPa.

The anisotropic property of porous material can be expressed by a semi-empirical equation^14^,^15^:


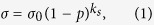


where *σ*_*0*_ is the strength of the nonporous material, *p* is the porosity, and *k*_*s*_ is a stress concentration factor that depends on the radius and height of the idealized cylindrical tubules in the porous material:


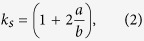


*a* and *b* are the traverse and longitudinal dimensions sections of the idealized cylindrical tubules. For stress application parallel to longitudinal direction of tubules, *b* approaches infinity and *k*_*s*_ = 1, while for stress perpendicular to the longitudinal direction of tubules, *a* *=* *b* and *k*_*s*_ = 3.

The measured porosity is 0.17. According to the stress concentration model, strength with loading perpendicular to the TP has a stress concentration *k*_*s*_ = 1 while on RP has a *k*_*s*_ = 3. The theoretical ratio of strengths (TP to RP ratio) is therefore 1.45. The experimental TP/RP ratio of average microhardness is 1.24 and the average compressive strength is 1.25. The difference between predicted and measured ratios may come from the non-ideal hexagonal distribution of cylindrical tubules and finite length of each tubule (this leads to *k*_*s*_ < 3 for RP). Therefore, the value of *k*_*s*_ shall be higher than 1 on the TP and less than 3 on the RP. This leads to a smaller experimental ratio than the predicted ratio. The analysis suggests that the fully dense material should have an elastic modulus larger than 2 GPa, which is closer to that of elephant ivory.

The fracture toughness (K, critical stress intensity factor, MPa·m^0.5^) is an important material property, and quantifies the ability of a material with a pre-existing crack to resist fracture. K values are influenced by intrinsic and extrinsic mechanisms^16^. The intrinsic mechanisms involve the inherent resistance of a material ahead of a crack tip, and determine K values for crack initiation. The extrinsic mechanisms operate at or behind the crack tip, and are correlated with shielding of the crack tip to reduce stress intensity (e.g. bridging). They depend on the size of the crack extension. The R-curve (K-crack propagation curve) is frequently used to characterize the fracture toughness of a material^16^,^17^,^18^. The schematic illustrations of flexural tests and sample orientations are shown in Fig. 2e.

The K-R curves for vegetable ivory are plotted in Fig. 2f and compared with those of elephant ivory and human cortical bone. SENB samples obtained from Jarina nuts are much smaller than their counterparts from elephant ivory. Therefore, to make the K-R curves comparable, a normalized crack extension Δa/b_0_ is used as the x-axis. The initial stress intensities of vegetable ivory are about 1 MPa·m^0.5^ for samples of both orientations, and are very similar to the initial stress intensities of elephant ivory and human cortical bone. The K-R curves of vegetable ivory do not present an obvious positive slope as those of elephant ivory. These results indicate that the vegetable ivory does not have significant extrinsic toughening mechanisms. The K values from samples in two orientations do not show significant difference.

Scanning electron micrographs of the crack extension (Fig. 3a,b,e,f) show that the cracks tend to propagate along the primary tubules and therefore path deflection occurs. This phenomenon is observed for samples of both orientations, and the deflection can be higher than 45 degrees due to the tendency to propagate through neighboring tubules (exemplified in Fig. 3b,f). The fracture surfaces for two orientations are illustrated in Fig. 3c,d,g,h. Due to crack deflection, they show a step-like morphology, which is very different from the flat regions ahead of the notches or pre-cracks. A schematic illustration of the fracture surface is shown in Fig. 3d,h. For orientation 1, because the tubules are not perfectly parallel to the crack plane but at an acute angle, the pre-crack region is relatively smooth and shows tilted ellipsoidal sections of the tubules. The step-like morphology of crack surface is the result of crack deflection. Each step represents a ring of primary tubules aligned in approximately one plane. The cracks tend to propagate along the longitudinal direction (along the primary tubules) because less energy is required.

The fracture surface of orientation 2 samples shows similar deflection and step-like morphology (shown in Fig. 3h). In contrast with orientation 1, we observe micro-cracks for orientation 2 along the longitudinal direction of tubules (near the main cracks). However, these cracks are also observed before the flexural tests (shown in Fig. 3e). Therefore, they are unlikely to be generated during the flexural tests and function as extrinsic toughening. On the samples, we do not observe any evidence of micro-cracking, and the tiny crack-like features are secondary tubules.

In contrast with dentin^16^, we do not observe uncracked ligaments or bridging structures that are considered as a major contribution to its toughness. Therefore, as the crack grows, crack deflection is the primary extrinsic toughening mechanism and does not result in a significantly increase in the critical stress intensity factor for vegetable ivory. This observation is consistent with the R-curve plot from mechanical testing, indicating that vegetable ivory does not possess the complex extrinsic toughening mechanisms of elephant ivory or bones.

In addition to its elegant appearance, carvability, and moldability with controllable drying process, vegetable ivory possesses outstanding mechanical properties. This material, especially taking the density into consideration, is competitive with elephant ivory and celluloid. Together with the appeals from environmental protection and sustainability it affords, vegetable ivory has the potential to substitute elephant ivory as well as several kinds of plastics for a broad range of applications like sculpture, buttons, handles, chopsticks, piano keyboards, etc.

Elephant ivory has cylindrical tubules that are embedded in a matrix formed by the mineralized collagen fibers; these are the result of odontoblast cell movement during dentin formation^16^,^17^. The collagen fibers that orient along the main axis of the tusk form a fabric reinforcement structure, with the organic phase occupying about 30% of its weight. The other 70% of its weight are hydroxyapatite-like crystals embedded in the organic matrix. The inorganic mineral provides the strength and the organic collagen provides the toughness^19^. Several significant extrinsic toughening mechanisms were observed by Kruzic^16^ as contributing to the fracture toughness of dentin: crack deflection, micro-cracking, crack bridging by collagen fibrils, and uncracked ligaments. Due to the presence of extrinsic toughening mechanisms, the fracture toughness of ivory shows a rising R-curve (see Fig. 2f).

Controlled solidification of the liquid Jarina endosperm extracted from either single or multiple seeds in molds could be used to manufacture the Jarina products with generic shapes and desired sizes. This proposed method is capable of producing uniform material with improved mechanical properties by eliminating the central lacuna due to rapid drying and the internal tubules. Therefore, in terms of manufacturing and processing, the vegetable ivory is superior to elephant ivory and is competitive with plastics.

Powder of vegetable ivory is a useful raw material for synthesis and fabrication. In this work, freeze casting, which has not been employed for biological/polymer powders to the authors’ knowledge, is used to fabricate porous scaffolds using micro-sized Jarina powder. These scaffolds are brittle and soft with a tactility that is similar to cotton (shown Fig. 3h). During the freezing, Jarina particles are placed and trapped between growing lamellar ice crystals and form a structure with interconnected ice channels. Once the ice is removed via sublimation through lyophilization, the freeze-cast scaffolds show pores on both transverse and longitudinal sections (shown in Fig. 3j,k). The Jarina scaffolds can be infiltrated with second phases to form a new composite material and to acquire varying/particular properties for multiple applications (e.g. toys, handles, keyboards).

In summary, the Jarina seed has a very pleasant appearance with comparable feel to the touch than the highly sought out properties of elephant ivory. However, the Jarina seed can be cultivated sustainably with processing methods that are competitive with plastics. This work presents original results on the microstructure and mechanical behavior of the Jarina seed, which include the following findings:

- It has a hexagonal lotus-type porous structure with a porosity of 0.17, and the primary cylindrical-like tubules arranged in rings and aligned in the radial direction.
- The anisotropic behavior of vegetable ivory results from the existence of tubules because: (a) no hierarchical structure is observed in the fracture surface; (b) its main component, hemicellulose, is amorphous; (c) nano-indentation without the interference of tubules does not show significant difference in hardness along the transverse and radial planes.
- While Jarina seeds have lower density, they show lower mechanical properties (Young’s modulus, 1.5 GPa, tensile strength, 26 MPa, and toughness, 0.65 MPa) than those of elephant ivory. Higher density seeds seem to be achievable by controlled processes.
- The anisotropic mechanical behavior of the Jarina seeds was studied using a spherical coordinate referential system that distinguishes the transverse (TP) and radial plane (RP); both microhardness and compressive strength in transversal plane are higher than those in the radial plane. Based on a stress concentration analysis, the elastic modulus ratio TP/ RP is 1.25, which validates this model, and further explains that the lower strength in RP is caused by stress concentration generation when stress is applied perpendicular to the cylindrical tubules.
- The K-R curve suggests that the initial fracture toughness of vegetable ivory (0.5–1.3 MPa.m^0.5^) is close to that of elephant ivory. The primary tubules play an important role as crack arresters, and the crack path deflection is observed as the major extrinsic toughening mechanism.

This sustainable material shows very good potential as an alternative to elephant ivory as well as to several other plastics, especially since no attempt has been made to enhance its mechanical properties artificially. Controlled solidification and freeze casting are suggested to process the raw material into intermediate goods with improved properties. In addition, it would be worthwhile to develop, through selective breeding, larger seeds, and tailored compositions in order to provide greater flexibility in manufacture. Processing of the hemicelluloses by introducing a mineral phase should increase the strength and match that of ivory, while retaining its favorable appearance. Future work should focus on depolymerizing, adding HAP crystals, and repolymerizing to obtain a novel bio-inspired material with superior mechanical properties.

## Methods

### Specimen preparation

The Jarina seed is only slightly soluble in water, so a water-assisted diamond saw was used to obtain the test samples from sun-dried bulk endosperm nuts. The samples are further dried at room temperatures for at least 24 hours before mechanical testing. Natural drying initiates from the surface of the nut to the center, so that in most seeds a lacuna is formed at the center; thus, all samples were obtained relatively far from the central lacuna.

### Microstructure characterization

X-ray micro-tomography was performed on a thin slice sample by Xradia MicroXCT-200 using 40 keV monochromatic X-rays. The voxel sizes are 5.0147 μm and 1.0197 μm, and the tomography data were reconstructed into 3-D images by XMReconstructor (Amira). In addition, an Axio-fluorescence microscope was employed to analyze samples prepared for mechanical testing. Fracture surfaces of the samples from fracture toughness tests were examined by a Phillips XL30 environmental scanning electron microscope (ESEM) and a FEI SFEG XL30 (UHR SEM) microscope to investigate the morphologies as well as the fracture mechanism. To avoid the unexpected growth of cracks under the vacuum chamber of SEM, critical point drying was performed using Tousimis AutoSamdri 815A. These samples were pre-coated with iridium by Emitech K575X Sputter Coater with a time interval of 7 seconds for SEM analysis.

### Tensile testing

The tests were carried out on INSTRON 3342 universal testing system equipped with a 500 N load cell at a strain rate of 10^−3^/s. The strains were measured by a SATEC strain gauge. Samples for tensile testing were cut and machined into a dog-bone shape using a steel mold. The dimensions are: whole length 15 ~ 25 mm, gauge length ~8 mm, width ~2 mm and thickness ~1.3 mm. Eight samples were used for statistical analysis of Young’s modulus, strength, and work to fracture.

### Micro and nanoindentation

Both the micro and the nanoindentation tests were conducted at room temperature. Sample sections were mounted in epoxy and polished. Vickers geometry 100 g and 200 g loads were applied with the LECOM400H1 Micro Hardness Test Machine and Hysitron nanoindentator. The microhardness values obtained represent the mean value of 10 indentations. Nanohardness test was carried out by a CSM Instruments Nanoindentation Tester (NHTX S/N: 0100005) with an indenter of Berkovich geometry (S/N BL09). The nano-indents avoid the tubules to eliminate their effects on the mechanical properties. The maximum load applied was 30 mN and the loading rate was 1 mN/s. The nanohardness value in this case is represented by the mean of 11 indentations.

### Compression testing

The compression tests were performed in an INSTRON 3367 at a strain rate of 10^−3^/s. The samples were cut into cubic shapes with side lengths of about 3 mm. At least 8 samples were tested to obtain statistically relevant values of the anisotropic mean elastic modulus, maximum strength, and toughness.

### Fracture toughness

Under linear-elastic conditions, a pre-existing crack will start to grow when the stress intensity at the crack tip exceeds the critical value, K (fracture toughness). We tested samples with two nominal crack growth directions: Orientation 1 in which loading is parallel to the transverse plane (TP), and Orientation 2 in which the loading is parallel to the radial plane (RP). The fracture toughness K was obtained from three-point bending tests on pre-cracked Single edge notch bending (SENB) samples using an INSTRON 3367 at a strain rate of 10^−3^/s. Based on the fracture toughness standards (ASTM E399 and E561) and the available equipment, we designed a sequential loading method, which records a series of points with crack length and flexural loads to obtain the R-curve This method can be summarized in five steps:

1. Three-point-flexural test is first carried out on one sample. Once the maximum load is reached, stop the test and unload the test sample. The decrease of the load beyond the maximum indicates the onset of propagation of Mode I (tension) crack.
2. The size of the crack is immediately measured with Axio-fluorescence microscope.
3. The sample is tested again after measuring the crack length.
4. Repeat steps 1, 2 and 3 for this sample 3, 5 or 7 times.
5. Analysis and calculation of K are performed for each flexure test (loading cycle) for each sample. Crack propagation path and fracture surface are observed using ESEM.

K is obtained from the following equation:


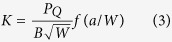


where *P*_*Q*_ is the critical load that is obtained using a 5% secant line (a line from origin with a slope equal to 95% of the initial elastic loading slope), and *f(a/W)* is a polynomial fit of *a/W* recommended in ASTM E 399:


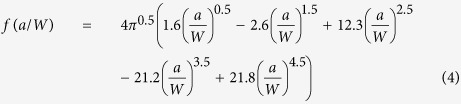


SENB samples for two orientations were prepared to verify possible anisotropy. The samples were machined with depth (W) ~ 7.5 mm, thickness (B) ~ 3 mm, and length ~ 30 mm (loading span (S) ~ 24 mm). An initial blunt notch was cut with a diamond blade and sharpened by a razor blade for each sample. The resulting notches had a length ~ 3 mm. The orientation of a sample was determined by observing the region near the notch tip using optical microscope.

### Freeze casting

First, micrometer-size Jarina powder was obtained through 24-hour alumina ball milling. Second, the powder is mixed with 1 wt% of polyethylene glycol, polyvinyl alcohol, and ammonium polymethacrylate anionic dispersant, Darvan 811 to form an aqueous slurry. Third, the slurry with 15 vol% of Jarina powder is frozen at a constant rate of approximately 10 K/min using liquid nitrogen controlled by a PID controller. After freezing, the porous scaffolds are generated through lyophilization using a Labconco freeze dryer at 233 K and 350 Pa for 72 hours. More details about theory and procedure of freeze casting can be found in literature^20^,^21^.

## Additional Information

**How to cite this article**: Chu, Y. *et al*. A Sustainable Substitute for Ivory: the Jarina Seed from the Amazon. *Sci. Rep*. **5**, 14387; doi: 10.1038/srep14387 (2015).

## Figures and Tables

**Figure 1:**
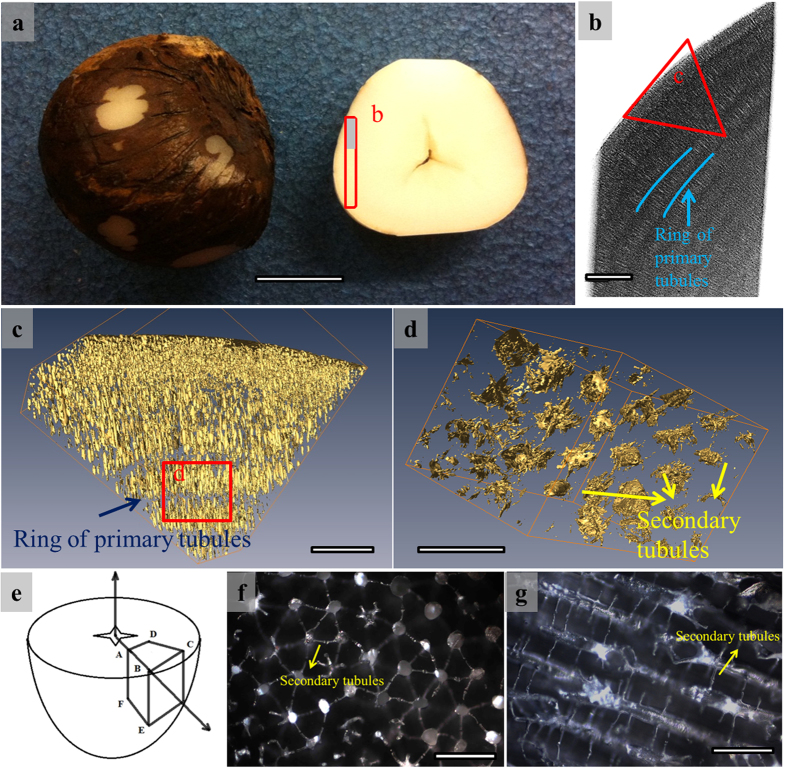
Overall structure of Jarina seed. (**a**) Overview and cross section of a Jarina seed; there is a central lacuna due to the shrinkage of natural drying process. Scale bar, 20 mm. (**b**) 3-D reconstructed X-ray tomography image of the area in red rectangle in (**a**). The tubules have relatively light color and are arranged in rings with their longitudinal axis perpendicular to rings (indicated by blue lines), while the solid area shows dark grey as background. Scale bar, 1 mm. (**c**) 3-D micro-CT image of the area in red triangle in (**b**) with a resolution of 5.0147 μm. The tubules are shown in golden color and the solid material is set to be transparent. Scale bar, 0.5 mm. (**d**) 3-D micro-CT image of the area in red rectangle in (**c**) with a resolution of 1.0197 μm. The near-hexagonal arrangement of the primary and secondary tubules can be observed. Scale bar, 0.1 mm. (**e**) Schematic diagram and definition of the directions; there is a lacuna located in the center of each seed which we define as the geometrical center of a spherical coordinate system. Plane BCE: transverse plane (TP) where we can observe the near hexagonal arrangement of the tubules in transverse section; Plane ABC/Plane ABE: radial plane (RP); (**f**) Optical micrograph taken from TP. Scale bar, 100 μm. (**g**) Optical micrograph taken from RP. Scale bar, 100 μm.

**Figure 2:**
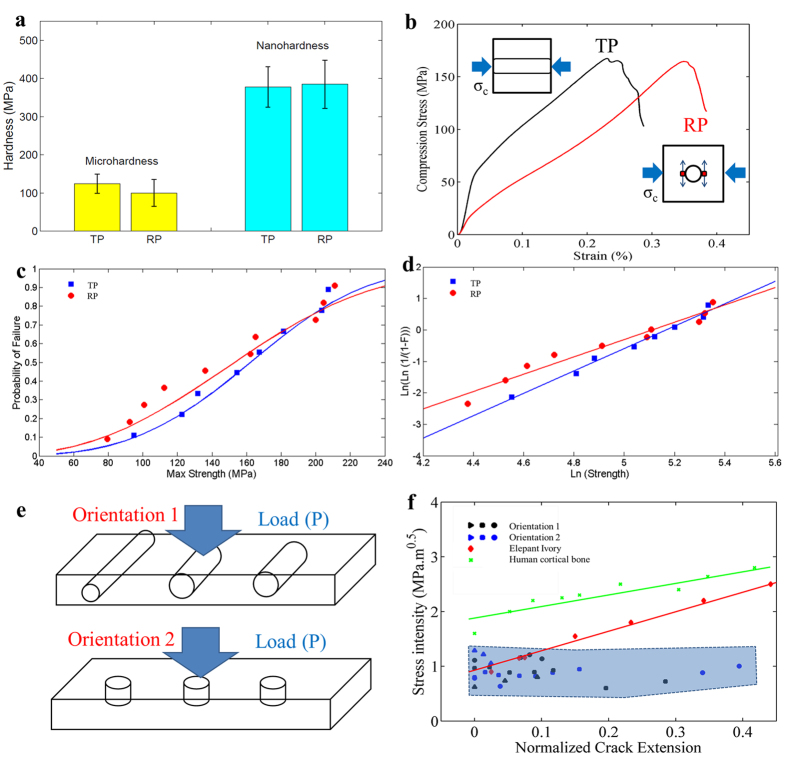
The mechanical response of Jarina seed. (**a**) Hardness histogram chart including micro and nano hardness values of Jarina seed from TP and RP. The error bars represent the 1.96 times the standard deviation. (**b**) Typical load-deformation curves of Jarina seed for two stress orientations: TP and RP. (**c**) Weibull distribution results from both TP and RP groups, the blue line and dots represent TP and the red line and dots represent RP. (**d**) Weibull plot to obtain m for TP (3.56) and for RP (2.76). (**e**) Schematic of the flexural testing in different orientations illustrated by the loading and the arrangement of cylindrical tubules. (**f**) K-R resistance curves for vegetable ivory (in shaded blue color), and representative K-R curves of elephant ivory^16^ and human corticalbone^18^ are also presented. Black and blue markers represent orientation 1 and 2, respectively.

**Figure 3:**
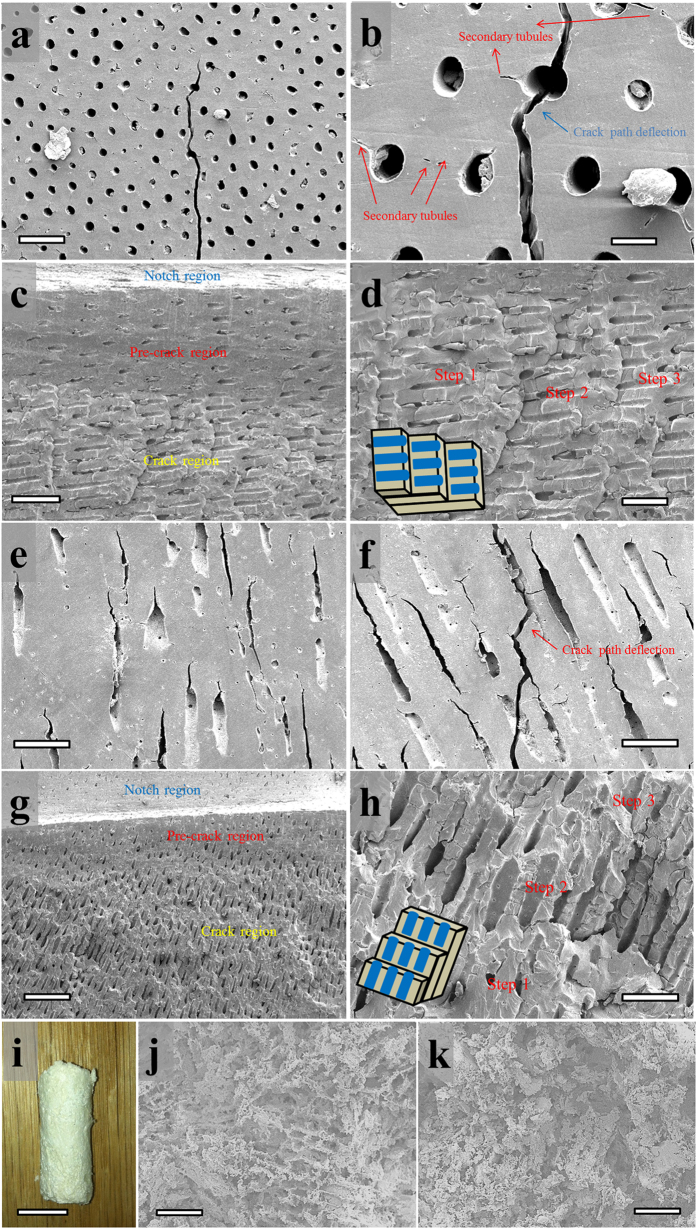
Crack propagation for fractured samples in orientations 1 (a-d) and 2 (e-h), and structure of a freeze-casted scaffold (i-k). (**a**) A representative crack path of orientation 1. Scale bar, 100 μm. (**b**) Deflection of crack path due to the existence of primary tubules. Scale bar, 25 μm. (**c**) Fracture surface showing the regions of notch, pre-crack and crack that are generated during flexural test. Scale bar, 100 μm. (**d**) Step-like morphology of the fracture surface. Scale bar, 50 μm. (**e**) Side view of a SENB sample of orientation 2 before flexural test. Scale bar, 100 μm. (**f**) Deflection of crack path due to the existence of primary tubules. Scale bar, 100 μm. (**g**) Fracture surface showing the regions of notch, pre-crack and crack that are generated during the flexural test. Scale bar, 250 μm. (**h**), Step-like morphology of the fracture surface. Scale bar, 100 μm. A schematic illustrating the step-like morphology observed from completely fracture SENB samples from orientation 1 and 2 is overlaid in (**d**,**h**), where the blue cylinders represent the tubules and are aligned in each step. (**i**), Sample of spherical freeze-cast scaffold. Scale bar 1 mm. (**j**), Transverse cross section of the spherical scaffold. Scale bar, 100 μm. (**k**), Longitudinal cross section of the spherical scaffold, Scale bar, 100 μm.

**Table 1:** Tensile Test Results^6^,^8^.

	Vegetable Ivory (Jarina)	Elephant Ivory (Dry)	Celluloid
Density (g/cm^3^)	1.2 ± 0.2	1.7~1.9	~1.4
Young’s Modulus (GPa)	1.5 ± 0.5	~12.5	1.38~1.73
Strength (MPa)	26 ± 10	36~110	39~47
Work to fracture (MPa)	0.65 ± 0.4	0.49~0.87	

The errors provided for vegetable ivory represent the 1.96 times the standard deviation.

**Table 2:** Micro Hardness.

Indentation Test	100 g (MPa)	200 g (MPa)
Vegetable Ivory (Jarina)		
—Transverse Plane	124 ± 25	129 ± 17
—Radial Plane	100 ± 35	103 ± 33
Elephant Ivory^11^		
—Circumferential Plane	390	350
—Radial Plane	310	270

The errors provided for vegetable ivory represent the 1.96 times the standard deviation. The elephant ivory data^11^ mention that their maximum standard error is less than 12%.

**Table 3:** Anisotropic Compression Test Results of Vegetable Ivory (Jarina).

Direction of Indentation	Tests Density (g/cm^3^)	Elastic Modulus (GPa)	Max Strength (MPa)	Strain to fracture	Work to fracture (MPa)
Transverse plane	1.20 ± 0.05	1.68 ± 0.7	158 ± 40	0.26 ± 0.06	25 ± 8
Radial Plane	1.20 ± 0.05	1.34 ± 0.4	146 ± 50	0.29 ± 0.07	27 ± 10

The errors provided for vegetable ivory represent the 1.96 times the standard deviation.
